# Phenotypic and Genotypic Analysis of Bacterial Pathogens Recovered from Patients Diagnosed with Fever of Unknown Origin in Egypt

**DOI:** 10.3390/antibiotics12081294

**Published:** 2023-08-07

**Authors:** Shimaa H. Mostafa, Sarra E. Saleh, Eman F. Khaleel, Rehab Mustafa Badi, Khaled M. Aboshanab, Samira M. Hamed

**Affiliations:** 1 Microbiology Lab Department, Abbasia Fever Hospital, Cairo 11566, Egypt; shimaa.hamdy08@pharma.asu.edu.eg; 2 Department of Microbiology and Immunology, Faculty of Pharmacy, Ain Shams University, Cairo 11566, Egypt; sarradeif@pharma.asu.edu.eg; 3 Department of Medical Physiology, College of Medicine, King Khalid University, Abha 61421, Saudi Arabia; eman_4000@hotmail.com (E.F.K.); rbadi@kku.edu.sa (R.M.B.); 4 Department of Microbiology and Immunology, Faculty of Pharmacy, October University for Modern Sciences and Arts (MSA), 6th of October, Giza 12451, Egypt; satwa@msa.edu.eg

**Keywords:** fever of unknown origin, Gram-negative pathogens, Gram-positive pathogens, multidrug resistance, priority pathogens

## Abstract

Fever of unknown origin (FUO) is a medical term describing fever that lasts for at least three weeks without a diagnosis being reached after extensive diagnostic evaluation. Therefore, this study aimed to identify the common pathogens causing FUO in patients admitted to Abbasia Fever Hospital in Egypt from January 2020 to December 2022, their antimicrobial susceptibility profiles, and associated resistance genes. The study also aimed to investigate the burden of multidrug-resistant (MDR) pathogens and the priority pathogens nominated by the World Health Organization (WHO) for posing the greatest threat to human health due to antibiotic resistance. During the study period, about 726 patients were diagnosed with FUO. After extensive investigations, the cause of the FUO was found to be infectious diseases in 479/726 patients (66.0%). Of them, 257 patients had positive bacterial cultures, including 202 Gram-negative isolates that comprised *Klebsiella pneumoniae* (85/202; 42.1%), *Escherichia coli* (71/202; 35.1%), *Acinetobacter baumannii* (26/202; 12.9%), and *Pseudomonas aeruginosa* (14/202; 6.9%) and 55 Gram-positive isolates, including *Staphylococcus aureus* (23/55; 41.8%), *Streptococcus pneumoniae* (7/55; 12.7%), and *Enterococcus* spp. (25/55; 45.5%). The MDR phenotype was shown by 68.3% and 65.5% of the Gram-negative and Gram-positive isolates, respectively. Carbapenem resistance (CR) was shown by 43.1% of the Gram-negative isolates. Of the 23 *S. aureus* isolates obtained from research participants, 15 (65.2%) were methicillin-resistant *S. aureus* (MRSA). A high-level aminoglycoside resistance (HLAR) phenotype was found in 52.0% of the *Enterococcus* sp. isolates. The PCR screening of resistance genes in the MDR isolates showed that *bla*_OXA−48_ was the most prevalent (84%) among the carbapenemase-coding genes, followed by *bla*_VIM_ (9%) and then *bla*_IMP_ (12%). The ESBL-coding genes *bla*_TEM_, *bla*_CTX-M,_ *aac(6′)-Ib*, and *bla*_SHV,_ were prevalent in 100%, 93.2%, 85,% and 53.4% of the MDR isolates, respectively. This study updates the range of bacteria that cause FUO and emphasizes the burden of multidrug resistance and priority infections in the region. The obtained data is of relevant medical importance for the implementation of evidence-based antimicrobial stewardship programs and tailoring existing empirical treatment guidelines.

## 1. Introduction

Fever of unknown origin (FUO) was defined as an illness of more than 3 weeks duration with a fever greater than 38.3 °C (101 °F) on several occasions, the cause of which is uncertain after 1 week of in-hospital investigations [1]. To keep with diagnostic capabilities, some modifications in the definition of FUO occurred throughout the years [1]. The results of the study conducted by Fusco et al. [2] carried out from 2005 to 2015 concluded that the most common causes of FUO are infectious diseases (37.8%), followed by non-infectious inflammatory diseases (20.9%), neoplasms (11.6%), and other diseases (6.5%), while the diagnosis remained unknown in 23.2% of cases [2]. Infectious diseases are the principal categories of diseases causing FUO, which may be caused by multidrug-resistant (MDR) bacterial infections [3].

MDR in bacteria is a great concern to humans’ and animals’ health and welfare [4]. Microbes may develop antimicrobial resistance after prolonged exposure to one or more antibiotics as a result of certain genetic mutations. In addition, the horizontal transfer of mobile genetic elements greatly contributes to the spread of antimicrobial resistance [5]. It has been reported that MDR bacterial infections kill around 50,000 individuals every year in the United States and Europe, and are estimated to kill more than 700,000 people worldwide [6]. If no action is taken to reduce MDR, 10 million people are predicted to die yearly from MDR infections by the year 2050 [6]. The World Health Organization (WHO) released a report titled “Microorganisms posing a substantial hazard to human health” that aimed to promote funding for the development of new antimicrobial drugs to counter the threat posed by a list of priority pathogens [7].

Gram-negative bacteria that are extensively drug-resistant (XDR) and carbapenem-resistant (CR) are thought to pose a worrying threat to human health worldwide [8,9,10,11]. The development of CR is currently gaining a lot of interest [8]. The excessive expression of class A, B, and D carbapenemase enzymes, such as *Klebsiella pneumoniae* carbapenemase (KPC), New Delhi Metallo-β-lactamase (NDM), imipenemase (IMP), and Verona Integron-encoded Metallo-β-lactamase (VIM) and oxacillinases (OXA−48) continues to be the most relevant route for the development of resistance among Gram-negative bacteria and has been responsible for the majority of nosocomial outbreaks in recent years [10,12]. In the current study, we sought to explore the contribution of bacterial infections in patients diagnosed with FUO in one of the major public fever hospitals in Egypt. In addition to defining the implicated bacterial species, we investigated the antimicrobial susceptibility of the bacterial isolates and explored the burden of MDR and WHO-priority pathogens. We extended our study to investigate some genetic determinants conferring resistance to clinically relevant classes of antimicrobial agents.

## 2. Results

### 2.1. Fever of Unknown Origin (FUO) Caused by Bacterial Infections

During the study period, from January 2020 to December 2022, about 726 patients were diagnosed with FUO. After extensive investigations, the cause of the FUO was found to be infectious diseases in 66.0% of cases, followed by returned travelers (9%), neoplastic diseases (4%), inflammatory diseases (2%), and other diseases (16%), and the diagnosis remained unknown in 2% of cases. The infectious disease cases of the FUO reached approximately 479/726 (66.0%). Of these, 257 positive bacterial cultures were obtained. These comprise 11.7% of the total number of specimens received by the hospital during the study period (*n* = 2200). The majority of positive bacterial cultures came from urine samples (*n* = 91, 35.4%), then blood (*n* = 82, 31.9%), sputum (*n* = 59, 22.9%), and cerebrospinal fluid (CSF) (*n* = 25, 9.72%).

**Figure 1:**
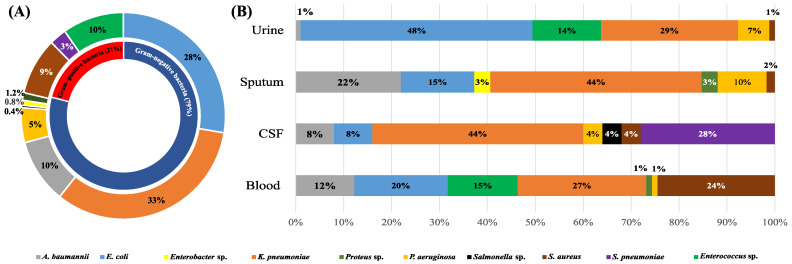
The spectrum of bacterial species collected in the current study (**A**) and their distribution in different specimen types (**B**). In (**A**), the percentage of each bacterial species was calculated with respect to the total number of isolates collected in the current study, while in (**B**), the percentage of each bacterial species was calculated with respect to the total number of isolates recovered from each specimen type.

A total of 55 Gram-positive isolates (20%) and 202 Gram-negative isolates (79%) were identified. The most-common Gram-positive species were *Staphylococcus aureus* and *Enterococcus* sp., whereas *Escherichia coli* and *Klebsiella pneumoniae* predominated among the recovered Gram-negative isolates. Figure 1 displays the full range of bacterial etiologies for FUO in the patient population included in the current study and the specimen distribution of various species. 

### 2.2. Antimicrobial Susceptibility Profiles

**Figure 2:**
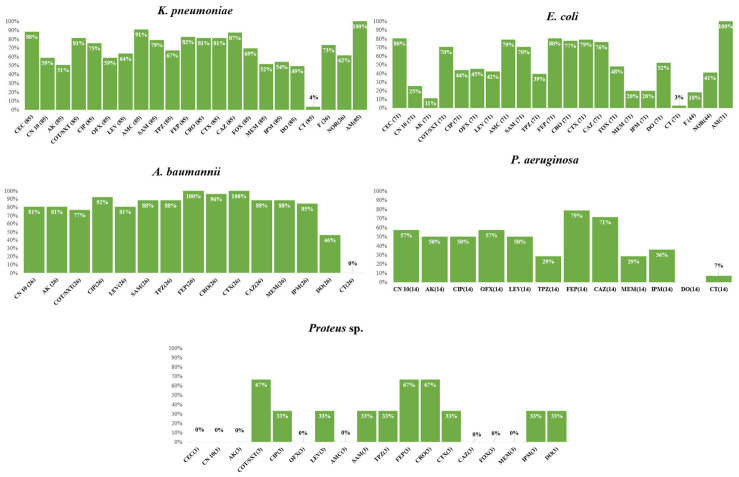
Frequency of antimicrobial resistance of various Gram-negative species to the tested antimicrobial agents. AMP, ampicillin; AMC, amoxicillin/clavulanic acid; AK, amikacin; CAZ, ceftazidime; CEC, cefaclor; CIP, ciprofloxacin; CN, gentamicin; TMP/SXT, trimethoprim–sulfamethoxazole; CRO, ceftriaxone; CT, colistin; CTX, cefotaxime; DO, doxycycline; FEP, cefepime; F, nitrofurantoin; FOX, cefoxitin; IPM, imipenem; MEM, meropenem; NOR, norfloxacin; OFX, ofloxacin; SAM, ampicillin/sulbactam; TPZ, piperacillin/tazobactam.

**Figure 3:**
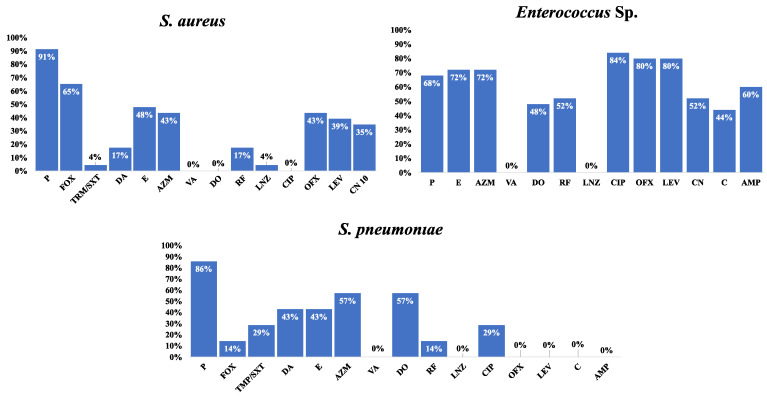
Frequency of antimicrobial resistance of various Gram-positive species to the tested antimicrobial agents. Cefoxitin is used as a surrogate for oxacillin disk-diffusion test; Susceptibility to vancomycin was tested using MIC determined by an E-test; Gentamicin 120 µg disk was used for testing HLAR is Enterococci; Oxacillin is used as surrogate for β-lactams disk-diffusion tests; Abbreviations: AMP, ampicillin; AZM, azithromycin; C, chloramphenicol; CIP, ciprofloxacin; CN, gentamicin; TMP/SXT, trimethoprim–sulfamethoxazole; DA, clindamycin; DO, doxycycline; E, erythromycin; FOX, cefoxitin; F, nitrofurantoin; LEV, levofloxacin; LNZ, linezolid; NOR, norfloxacin; OFX, ofloxacin; P, penicillin; RF, rifampin; VA, vancomycin. Percentages were calculated with reference to the total number of the tested isolates.

The frequency of the antimicrobial resistance of various species to the studied antimicrobial drugs in Gram-negative and Gram-positive species are shown in Figure 2 and Figure 3, respectively. Only one *Salmonella* spp. isolate was included in the study. This was susceptible to cefaclor, gentamicin, amikacin, trimethoprim–sulfamethoxazole, ciprofloxacin, ofloxacin, ampicillin/sulbactam, piperacillin/tazobactam, cefepime, ceftriaxone, cefotaxime, ceftazidime, cefoxitin, imipenem, and meropenem. The isolate was non-susceptible only to amoxicillin-clavulanic acid and doxycycline. Interestingly, we found a significant association between non-susceptibility to some antimicrobial agents, as shown in Figures S1–S8.

The MDR phenotype was shown in 138/202 (68.31%) of the Gram-negative isolates. The MDR phenotype was most frequently shown by *A. baumannii* (23/26; 88.46%), *E. coli* (40/71; 56.33%), and *Klebsiella pneumoniae* (69/85; 81.17%). Some genera had a lower prevalence of MDR, such as *P. aeruginosa* (6/14; 42.85%). None of the isolates that belonged to the *Enterobacter* species and *Proteus* species showed the MDR phenotype. Of the Gram-positive isolates, only 65% (36/55) were MDR. These comprised *Enterococcus* sp. (19/25; 76.00%), *S. pneumoniae* (5/7; 71.42%), and finally *S. aureus* (12/23; 52.17%). Carbapenem resistance (CR) was shown by 43.06% of the Gram-negative isolates. Glucose non-fermenters had a higher prevalence of CR (27/40; 67.50%) compared to *Enterobacteriaceae* isolates (60/162; 37.03%). The three species with the highest rates of CR were *A. baumannii* (22/26; 84.61%), *K. pneumoniae* (46/85; 54.11%), and *P. aeruginosa* (6/14; 42.85%). Only 19.71% (14/71) of the *E. coli* isolates were CR.

Associations were found between the resistance phenotypes and more antimicrobial agents in Gram-negative species, as shown in Figure S1–S5. Moreover, the association between the resistance phenotypes to different antimicrobial agents showed a statistically significant association between resistance phenotypes to cefoxitin, levofloxacin, and gentamicin in *S. aureus*; chloramphenicol, ofloxacin, levofloxacin, and gentamicin in the *Enterococci* (Figure S6–S8).

Of the 23 *S. aureus* isolates obtained from research participants, 15 (65%) were methicillin-resistant *S. aureus* (MRSA), while all were susceptible to vancomycin (MIC range = 0.125–2 µg/mL). Thirteen isolates of *Enterococcus* sp. underwent high-level aminoglycoside resistance (HLAR) testing. The HLAR phenotype was found in 52% of all *Enterococcus* sp. isolates. In 86% of *S. pneumoniae* isolates, penicillin resistance was phenotypically evident. In the meantime, the E-test demonstrated that all *S. pneumoniae* isolates were sensitive to cefotaxime, ceftriaxone, or imipenem.

**Figure 4:**
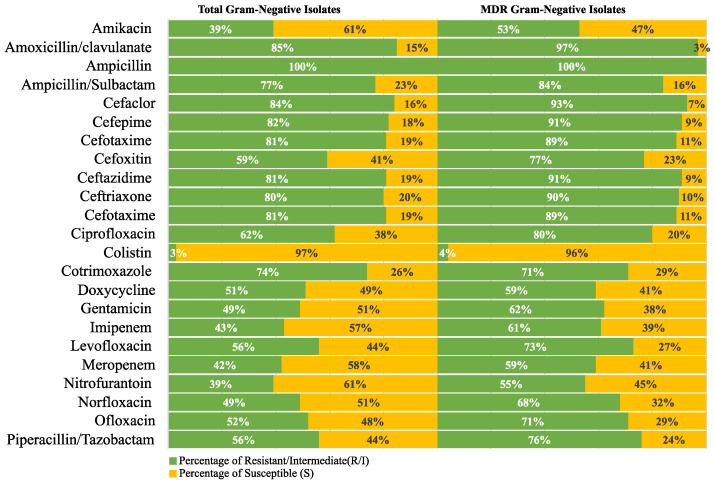
Resistance frequency of the tested antimicrobial agents in all MDR Gram-negative isolates.

**Figure 5:**
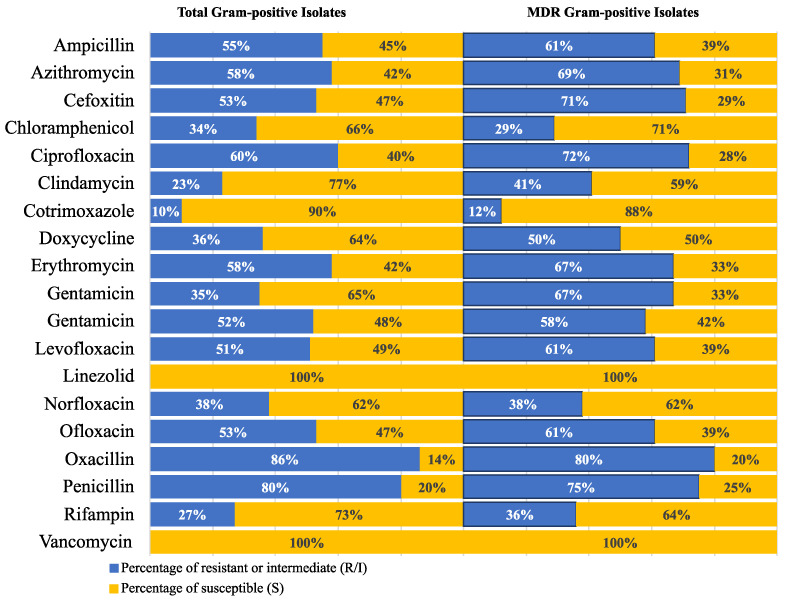
Resistance frequency of the tested antimicrobial agents in all MDR Gram-positive isolates.

The resistance frequency for the tested antimicrobial drugs in all Gram-negative and Gram-positive isolates was examined to provide more helpful information for directing empirical treatment guidelines. A similar analysis was conducted on the MDR isolates. The analysis findings are displayed in Figure 4 and Figure 5.

### 2.3. Minimum Inhibitory Concentrations of the Tested Antibiotics

One hundred and three Gram-negative isolates showing the MDR phenotype were selected for the broth microdilution assays for the determination of the minimum inhibitory concentrations (MICs) of selected antimicrobial agents and correlating the MICs with some antimicrobial resistance genes. The MDR isolates selected for further analysis included 53 *K. pneumoniae,* 26 *E. coli,* 20 *A. baumannii,* and 4 *P. aeruginosa* isolates.

The MIC of imipenem ranged from 4 to 256 µg/mL with an MIC_50_ of 64 μg/mL, but the MIC of cefepime ranged from 24 to 512 µg/mL with an MIC_50_ of 256 µg/mL. On the other hand, the MIC of cefotaxime ranged from 24 to 512 µg/mL with an MIC_50_ of 512 µg/mL, and finally, the MIC of ciprofloxacin ranged from 6 to 512 µg/mL with an MIC_50_ of 128 µg/mL. All isolates were found to be susceptible to colistin by E-test. The data summary of the MICs is shown in Table S1.

### 2.4. Molecular Detection of the Carbapenemase-, Extended-Spectrum β-Lactamase (ESBL)-, and aac(6′)-Ib-Coding Genes

**Figure 6:**
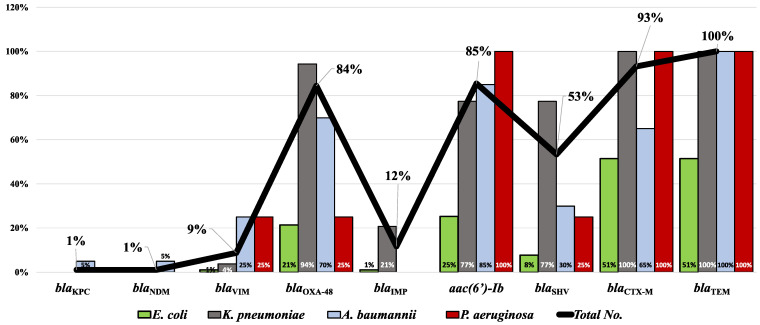
Prevalence of the screened antimicrobial resistance genes in the tested MDR clinical isolates. Bars represent the percentage of each gene in each bacterial species while the line represents the total prevalence of each gene.

Of all the carbapenemase-coding genes screened here, *bla*_OXA−48_ was the most prevalent and was carried by 87 (84%) isolates, followed by *bla*_VIM_, which was detected in 9 (9%) isolates, and *bla*_IMP_ was observed in only 12 (12%) isolates. The carbapenemase-coding genes *bla*_KPC_ *and bla*_NDM−1_ were the least prevalent; each was identified in one *A. baumannii* isolate. Screening our isolates for the ESBL-encoding genes showed that the predominant gene was *bla*_TEM_ (100%), then *bla*_CTX-M_ (93.2%), and the least prevalent was *bla*_SHV_ (53.4%). Finally, the *aac(6′)-Ib* gene was found in a relatively high prevalence, reaching 85%. The total prevalence of all genes as well as their distribution in different species are shown in Figure 6.

Some isolates were found to carry CR genes while retaining phenotypic susceptibility to carbapenem antibiotics. Among *E. coli*, carbapenem susceptibility was retained by 12 isolates carrying *bla*_OXA−48_ and two isolates that carried at least one of the CR genes *bla*_VIM_ or *bla*_IMP_. In *K. pneumoniae*, ten isolates carried *bla*_OXA−48_ as a single gene and two isolates had three combined genes (*bla*_OXA−48_, *bla*_VIM,_ and *bla*_IMP_). Interestingly, all were susceptible to the tested carbapenems. In *A. baumannii*, only one carbapenem-susceptible isolate was found to carry the *bla*_VIM_ gene.

A data summary of the antimicrobial resistance genes carried by different species correlated to the MICs of imipenem, cefepime, cefoxitin, and ciprofloxacin is shown in Table S1.

**Figure 7:**
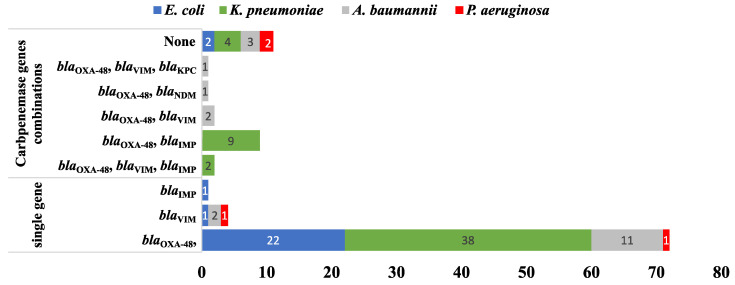
A bar chart summarizing the number of CR genes carried by the tested MDR isolates (*n* = 103) harboring CPases-encoding genes either in combination or as single genes.

Remarkably, 15/103 (14.56%) MDR isolates co-harbored more than one CPase-coding gene. In comparison, 77/103 (74.75%) carried only a single gene (Figure 7). Out of the 103 isolates harboring CPases-encoding genes, the co-existence of *bla*_OXA−48_ and *bla*_IMP_ was detected in nine isolates (8.7%) while both (*bla*_OXA−48_ and *bla*_VIM_) and (*bla*_OXA−48_, *bla*_VIM_ and *bla*_IMP_) co-existed in two isolates for each. Finally, (*bla*_OXA−48_ and *bla*_NDM_) and (*bla*_OXA−48_, *bla*_KPC_ and *bla*_VIM_) were recorded in one isolate (0. 97%). Testing the association between the resistance genes and the phenotypic resistance to different antimicrobial agents showed that only *bla*_VIM_ had a statistically significant association with imipenem and meropenem resistance, with *p*-values of 0.011 and 0.026, respectively. This was only found among *K. pneumoniae* isolates. None of the ESBL-coding genes showed a statistically significant association with resistance to any of the tested β-lactam antibiotics. Finally, *acc-(6′)-Ib* showed a statistically significant association with gentamicin resistance (*p*-value 0.045) in *A. baumannii* but not with amikacin or any of the tested fluoroquinolones. The *p*-values calculated for all genes and antimicrobial agents are shown in Tables S2–S4.

### 2.5. Phenotypic and Genotypic Analysis Using Heatmap Analysis

**Figure 8:**
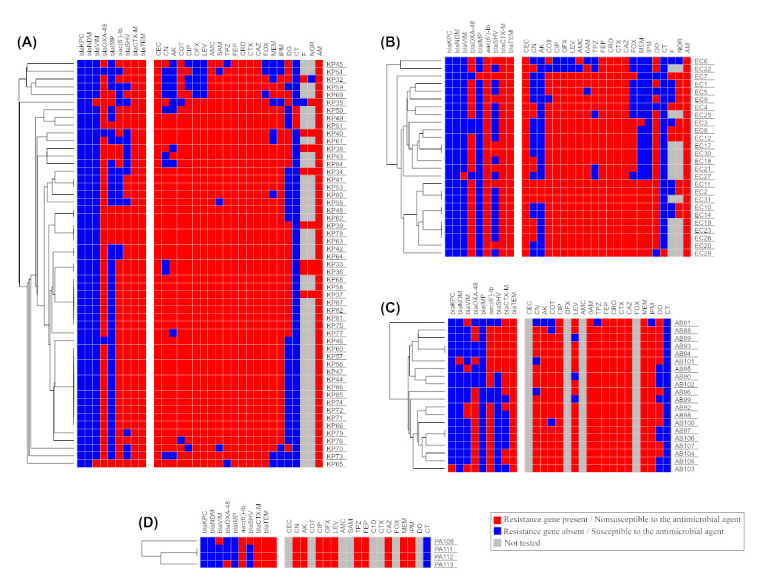
Heatmap analysis (**A**) for 53 isolates from *K. pneumoniae,* (**B**) for 26 isolates from *E. coli*, (**C**) for 20 isolates from *A. baumannii*, (**D**) for 4 isolates from *P. aeruginosa.* The figure also shows a hierarchical clustering for all isolates that belong to the same species based on the available phenotypic and genotypic data.

Using the antimicrobial resistance profiles, carbapenemase genes, ESBL genes, and *aac-(6′)-Ib* gene results, a dendrogram depicting the heatmap signature of the isolates was produced. The 53, 26, 20, and 4 MDR *K. pneumoniae, E. coli, A. baumannii,* and *P. aeruginosa* isolates were clustered into 31, 21, 18, and 3 clusters, respectively (Figure 8).

## 3. Discussion

This is a two-year study conducted in the Abbasia Fever Hospital, a major infectious disease public hospital serving the area of Greater Cairo in Egypt. The study provides an update on the recent spectrum of bacterial pathogens causing FUO and their susceptibility profiles. Upon culturing 726 clinical specimens received by the hospital’s laboratory for the investigation of FUO, only 257 (35.4%) showed bacterial growth. Other infectious causes of FUO (30.6%) may include unculturable bacteria and those diagnosed principally through serological tests such as *Salmonella* sp. and *Brucella* sp. They may also include viruses, fungi, or parasites. These are typically underdiagnosed since they are primarily diagnosed through serological, molecular, or histological testing, which may not be practical in many hospitals, especially in nations with poor healthcare resources [13]. Reports on the prevalence and etiology of the FUO in Egypt are relatively scarce. FUO with an infectious etiology prevailed in most of the previous studies [14,15,16,17], with a prevalence that reached 72% [14]. In a study by Montasser et al. [17] conducted in 2015 on patients admitted to Abbassia Fever Hospital with FUO, cytomegalovirus infection was identified as the most common infectious cause, followed by urinary tract infections and respiratory tract infections. Less frequently, the infectious causes of the FUO included salmonellosis, brucellosis, tuberculosis, and infective endocarditis. In the same study, the Gram-negative isolates were predominated by *E. coli*, *Klebsiella* sp., and *Enterobacter* sp., while *S. aureus* was the most common Gram-positive species.

The bacterial isolates that were recovered from the patients enrolled in our study included 202 Gram-negative and 55 Gram-positive isolates. Compared to our previous study conducted in 2018 and 2019 [18], a lower percentage of positive bacterial cultures were found among the total number of specimens received by the microbiology laboratory of the Abbasia Fever Hosiptal in Cairo (24.7% versus 11.7%). This is likely due to the contribution of other viral infections such as COVID-19 that emerged early in 2020. A considerable share was found for the MDR isolates in the current study. Up to 67.7% of all isolates were MDR. This comprised 138/202 (68.3%) and 36/55 (65.5%) of the Gram-negative and Gram-positive isolates, respectively. The high prevalence of MDR strains in hospital settings has been widely reported in Egypt [19,20,21,22,23] and in other countries as well [24,25,26]. A further rise in the prevalence of the MDR strains was also evident during the pandemic of COVID-19 [23,26,27,28]. In FUO, MDR infections have been recently linked to prior colonization by MDR organisms that were in turn linked to a longer hospitalization [29].

Of 162 *Enterobacteriaceae* isolates investigated here, 109 (67.28%) showed MDR. This was most frequently found in *K. pneumoniae* (69/85; 81.17%) and *E. coli* (40/71; 56.33%) isolates. Yet, a higher prevalence of MDR (84.6%) was shown by *Acinetobacter baumannii* isolates. Carbapenems are among the last-resort treatment options for severe Gram-negative infections, particularly those that are MDR. As reported by El-Kholy et al. [19], *A. baumannii*, *K. pneumoniae*, and *P. aeruginosa* are among the top-ranked CR Gram-negative species in Egypt. At least 37% of *Enterobacteriaceae* isolates were non-susceptible to at least one carbapenem. Interestingly, 54% of the carbapenem-resistant *Enterobacteriaceae* belonged to *K. pneumoniae*. Other carbapenem-resistant isolates comprised *E. coli* (20%). Carbapenem-resistant *K. pneumoniae* (CRKP) is well-recognized as a leading cause of hospital-acquired infections worldwide [30] as well as in Egypt [31,32,33]. The susceptibility profiles of the ESBL producers were encountered in 79% of *Enterobacteriaceae* isolates. More than half of *K. pneumoniae* (70/85; 82%) isolates were predicted to be ESBL producers. Similar findings were reported by others [34]. Carbapenem-resistant *A. baumannii* (CRAB) is among the high-priority pathogens listed by the WHO. In our collection, CRAB was evident in 85% of the *A. baumannii* isolates. The wide dissemination of carbapenem-resistant *Acinetobacter baumannii* (CRAB) was previously reported by regional [35,36,37] and worldwide studies [38,39]. CRAB was also identified in a higher prevalence in some recent studies from Egypt [40]. Therapeutic options for the treatment of CRAB infections are limited to colistin, tigecycline, and some aminoglycosides [39].

MRSA is another high-priority pathogen presented here. A total of 23 *S. aureus* infections were found; 15/23 (65.2%) of the isolates were resistant to penicillinase-stable penicillins, and 52.17% were MDR. In comparison to other African [41] and Mediterranean [42] nations, previous investigations have indicated that Egypt has the highest MRSA scores. Vancomycin has long been acknowledged as the cornerstone of MRSA infection treatment [43]. Fortunately, none of the MRSA isolates were resistant to vancomycin.

Five out of seven (71.42%) *S. pneumoniae* isolates identified here showed the MDR phenotype. Nevertheless, medium priority was assigned by the WHO for penicillin-resistant *S. pneumoniae*. Using the oxacillin disk-diffusion test, penicillin resistance was inferred in 6/7 (85.7%) *S. pneumoniae* isolates. Of them, seven isolates were tested for susceptibility to cefotaxime, ceftriaxone, or imipenem using an E-test, as recommended by the Clinical and Laboratory Standards Institute (CLSI) guidelines [44]. All the isolates (100%) were susceptible to all of the tested agents. In contrast to our findings, a prevalence of 49.0% of penicillin resistance was reported by Wasfy et al. [45] in *S. pneumoniae* isolates collected between 1993 and 2003 in Egypt. Furthermore, susceptibility to penicillin was maintained in 84.2% of *S. pneumoniae* isolates tested by El-Kholy et al. in a more recent study [46]. The high prevalence of penicillin resistance identified here was balanced by acceptable levels of susceptibility to other antimicrobial classes, highlighting the critical need for empirical treatment regimen re-evaluation.

The human gut inhabitants, *Enterococci,* are well-recognized as challenging nosocomial pathogens frequently causing difficult-to-treat infections [47]. MDR *Enterococci* comprised 76% of the *Enterococcal* isolates investigated here. The combination therapy of cell-wall inhibitors with aminoglycosides has long been used for the treatment of serious enterococcal illnesses [48]. The effectiveness of this treatment regimen is eliminated by the reduced susceptibility to either of the combined antimicrobials. Hence, synergy is routinely predicted using the HLAR test. The test revealed high-level resistance to aminoglycosides in 13/25 (52.0%) *Enterococcal* isolates in our collection.

Investigating the association between the resistance phenotypes to different antimicrobial agents showed a statistically significant association between the resistance phenotypes to some antimicrobial agents. This was mostly found between antimicrobial agents belonging to the same class, which are likely affected by the same resistance determinants. Occasionally, a statistical significance was found between antimicrobial agents that belong to different classes. This is likely due to the co-existence of some resistance genes on the same plasmids or other mobile genetic elements [49,50]. It is worth mentioning that such associations were more frequently found among Gram-negative species (Figure S1–S8).

One of the goals of our study was to investigate the contribution of different classes of CPases to the CR phenotype shown by the MDR isolates identified here. For this purpose, we have molecularly characterized the carbapenemase-encoding genes in a group of MDR isolates.

The *bla_OXA−48_* gene was the predominant carbapeneamse-coding gene, found in 84.5% of the isolates, followed by *bla*_IMP_ (10.7%), *bla*_VIM_ (8.7%), *bla*_NDM_ (1%), and *bla*_KPC_ (1%). According to Abdelaziz et al. [51], *bla*_NDM_ (80.5%) was the predominant CR gene, followed by *bla*_VIM_ (36.4%), *bla*_KPC_ (28.6%), *bla*_OXA−48_ (26%) and *bla*_IMP_ (6.5%]. Imipenem MIC_50_ values were 64, with MIC values ranging from 4 to 512 µg/mL. It is worth mentioning that *bla*_NDM_ and *bla*_KPC_ were exclusively found in *A. baumannii*. While *bla*_NDM_ was frequently reported in *A. baumannii* [11,36,37,40], only a few reports about KPC-producer *A. baumannii* have been published [37,52,53]. Combinations of up to three CR genes were detected in our collection. The most frequently detected combination was *bla*_OXA−48_ and *bla*_IMP_, which was exclusively carried by *K. pneumoniae* isolates. No combinations of CR genes were found in *E. coli* or *P. aeruginosa*. Many studies have also noticed the co-existence of carbapenemase-coding genes, which resulted in decreased sensitivity to various antibiotics [49,54,55,56]. Interestingly, some of our isolates that carried one or more of the CR genes (*bla*_KPC_, *bla*_IMP_, *bla*_VIM_, or *bla*_NDM_) showed phenotypic susceptibility to imipenem. These results contrasted with other studies confirming that MBL genes confer phenotypic resistance to carbapenems [56,57]. In line with our findings, carbapenem-susceptible strains carrying CR genes were also reported by other authors [55,58,59,60,61]. Kayama et al. proposed that some carbapenemases may affect meropenem but not imipenem [62].

Extended-spectrum β-lactamases (ESBLs) are one of the most clinically significant subgroups of β-lactamases [63]. Numerous investigations have shown that patients who are infected with bacteria that encode ESBLs have higher mortality rates and, generally, worse clinical outcomes [63]. More than half of the isolates studied here carried at least one of the ESBL-coding genes *bla*_SHV_*, bla*_CTX-M_, and *bla*_TEM_. This was slightly higher than the percentage found in our previous study conducted in 2018 and 2019 [18]. ESBL-coding genes were found in combinations in the majority of the isolates, most frequently in *K. pneumoniae* (Table S1). Also, cefotaxime and cefepime MIC_50_ values were 512 and 256, respectively, with MIC values ranging from 24 to 512 µg/mL for both. No statistical significance was found between any of the tested ESBL genes and the resistance phenotypes of β-lactams. This was partly due to the high fraction of the isolates that were non-susceptible to most of the tested β-lactams. Moreover, all isolates were positive for *bla*_TEM_. Hence, the *p*-values could not be calculated in many cases, as shown in Tables S2–S4.

A major issue in the therapeutic management of infections is the concurrent quinolone resistance in ESBL-producing *Enterobacteriaceae*. The fluoroquinolone-acetylating aminoglycoside-(6′)-N-acetyltransferase (*aac(6′)-Ib-cr*) gene is one of the most frequently identified plasmid-mediated quinolone resistance (PMQR) determinants. The gene is commonly found to co-exist with other PMQR determinants, including *qnr* genes [64,65,66]. A remarkably high frequency of fluoroquinolone resistance was found in the current investigation. Such a high FQ resistance was accompanied by the existence of *aac(6′)-Ib* determinants in 85.4% of the clinical isolates. An MIC_50_ of 128 µg/mL was recorded for ciprofloxacin in our isolates, with MIC values ranging from 6 to 512 µg/mL. Nevertheless, the association between the *aac(6′)-Ib* gene and ciprofloxacin resistance was not significant in our isolates. This is likely because *aac(6′)-Ib-cr* is not sufficient to confer ciprofloxacin resistance. As reported by Hamed et al. [65], full resistance to ciprofloxacin occurs due to multiple mechanisms, among which target site alteration is the main player.

The clonal relatedness of the MDR isolates studied here was investigated by a hierarchical clustering based on both the genotypes and phenotypes. The analysis showed that most of the isolates were not clonal, reflecting that the screened genes were likely disseminated by horizontal transfer rather than vertically by clonal expansion (Figure 8).

Taken together, the results of the current study highlight the serious spread of MDR organisms in the community and their implications for FUO, whose definitive diagnosis is particularly challenging in the outpatient setting. In an attempt to contribute to developing treatment guidelines tailored to the currently circulating MDR strains, the antimicrobial susceptibility findings of the current study were used for ranking the tested antimicrobial agents based on their activity against all isolates as well as those showing MDR. The top-ranked antimicrobials that showed the highest activity against Gram-negative isolates were colistin, amikacin, and nitrofurantoin. However, one of the limitations of our study is that the colistin susceptibility of the isolates was tested using an E-test that was reported before to underestimate colistin MICs, resulting in a significant number of false-susceptible results [67,68,69]. A similar analysis was performed for Gram-positive isolates, and the top-ranked antimicrobials included vancomycin, linezolid, co-trimoxazole, chloramphenicol, and rifampicin. All showed acceptable activity against MDR Gram-positive isolates. Accordingly, our findings support other calls for reviving interest in older antimicrobials for the management of infections caused by MDR bacterial strains [70,71,72]. In addition, several alternative approaches have also been proposed by many researchers for managing MDR infections. Among others, these include antimicrobial combination therapy [73], bacteriophage therapy [74], and nanoparticles [73]. The proper implementation of antimicrobial stewardship and infection control programs is also crucial for reducing the emergence and spread of MDR strains [74].

## 4. Materials and Methods

### 4.1. Study Design

The current study is a prospective study conducted at Abbasia Fever Hospital in Cairo in the period from January 2020 to December 2022. The Abbassia Fever Hospital is one of the largest infectious disease hospitals in Egypt. It is affiliated with the Egyptian Ministry of Health, and all febrile patients from the Greater Cairo Area are referred there. The study protocol was in agreement with the ethical principles stated in the Declaration of Helsinki and was approved by the institutional ethics committee, Faculty of Pharmacy, Ain Shams University (ENREC-ASU-2019-268).

### 4.2. Microbiological Procedures

During the study period, about 2200 clinical specimens, including urine, blood, sputum, and cerebrospinal fluid (CSF) were received by the microbiology laboratory of the hospital. The specimens were collected from patients admitted to Abbassia Fever Hospital in 2020 and 2021 for investigation of the cause and etiology of FUO after a failure to establish a definitive diagnosis in the outpatient setting. Of them, positive bacterial cultures were obtained from 257 cases selected for enrollment in our study. The isolates were recovered from various clinical specimens obtained from febrile neutrophilic patients (>11,000 white blood cells/µL with oral temperature >38 °C, over at least 3 days for inpatient or at least 3 weeks for outpatient). Bacterial isolates were identified routinely by morphological and biochemical tests and supplemented as needed by specialized tests and special agar media, according to the guidelines of the Central Health Laboratories affiliated with the Egyptian Ministry of Health. Blood specimens were cultured in trypticase soya broth blood culture vials that were then incubated at 35–37 °C in a non-CO_2_ incubator for a maximum of 14–21 days. Subcultures were made on blood agar, chocolate agar, and McConkey agar for subsequent identification. Pathogens in CSF specimens were identified by Gram staining, and cultures were made on chocolate agar, blood agar, nutrient agar, and MacConkey agar after incubation at 37 °C for 24 h. Blood and chocolate agar plates were incubated in 5–10% CO_2_ conditions. Urine specimens were cultured on nutrient (or blood agar) and MacConkey agar or CLED (cystine-lactose-electrolyte-deficient) agar plates that were then incubated at 35 °C for 24 h. Sputum specimens were cultured on blood, chocolate, and MacConkey agar plates. Incubation conditions were aerobic for 24 h at 35 °C for MacConkey agar plates and 5–10% CO_2_ for blood and chocolate agar. The identification of the isolates that exhibited the MDR phenotype was confirmed by the VITEK2 automated system (bioMérieux, Marcy L’Etoile, France).

### 4.3. Antimicrobial Susceptibility Testing

The antimicrobial susceptibility testing of recovered isolates was carried out according to CLSI (2021) for each bacterial species [44]. The Kirby–Bauer disk-diffusion method was performed on Müller–Hinton agar (Hi media, Maharashtra, India) using the following antimicrobial disks (Bioanalyse, Ankara, Turkey): penicillin (P, 10 U), ampicillin (AMP, 10 μg), amoxicillin/clavulanic acid (AMC, 20/10 μg), ampicillin/sulbactam (SAM, 10/10 μg), piperacillin/tazobactam (TPZ, 10/100 μg), cefaclor (CEC, 30 μg), cefoxitin (FOX, 30 μg), cefepime (FEP, 30 μg), ceftriaxone (CRO, 30 μg), cefotaxime (CTX, 30 μg), ceftazidime (CAZ, 30 μg), imipenem (IMP, 10 μg), meropenem (MEM, 10 μg), gentamicin (CN, 10 μg and 120 μg) amikacin (AK, 30 μg), trimethoprim-sulfamethoxazole (cotrimoxazole) (TMP/SMX, 1.25/23.75 μg), ciprofloxacin (CIP, 5 μg), levofloxacin (LEV, 5 μg), norfloxacin (NOR, 10 μg), ofloxacin (OFX, 5 μg), doxycycline (DO, 30 μg), nitrofurantoin (F, 300 μg), erythromycin (E, 15 μg), azithromycin (AZM, 15 μg), chloramphenicol (C, 30 μg), clindamycin (DA, 2 μg), vancomycin (V, 30 μg), rifampin (R, 5 μg) and linezolid (LNZ, 30 μg). Susceptibility to colistin was examined using an E-test (Bioanalyse, Turkey) according to the manufacturer’s recommendations. Both *E. coli* ATCC 25922 and *S. aureus* ATCC^®^ 25923 reference strains were used for the quality control.

The following issues were considered during antimicrobial susceptibility testing: (1) cefoxitin was used as a surrogate for oxacillin in the disk-diffusion test for the identification of methicillin (oxacillin)-resistant *Staphylococci* (MRS); (2) MRSA was considered resistant to all other β-lactams except cephalosporins with anti-MRSA activity (ceftaroline and ceftobiprole); (3) An E-test (Bioanalyse, Turkey) was used for testing the susceptibility of *Staphylococci* to vancomycin; (4) Oxacillin was used as a surrogate for β-lactams disk-diffusion tests in *S. pneumoniae*. (5) *S. pneumoniae* isolates with oxacillin zone diameters of ≤19 mm were tested for susceptibility to cefotaxime, ceftriaxone, or imipenem using an E-test (Bioanalyse, Turkey) when possible; and (6) HLAR in *Enterococci* was tested by disk-diffusion test using 120 µg gentamicin disks. The minimum inhibitory concentrations of some selected antimicrobial agents in the MDR Gram-negative isolates were determined using the broth microdilution assay/E-test according to the recommendations of the CLSI guidelines [44].

### 4.4. Identification of MDR and ESBL Phenotypes

The MDR phenotype was identified as previously reported by Magiorakos et al., who defined it as resistance to a minimum of one antimicrobial agent in three or more categories of antimicrobials [75].

For identifying the ESBL production phenotype, a modified version of the Jarlier double-disk synergy (DDS) approach was applied as recommended by the CLSI [44]. A disc containing amoxicillin (20 mg) and clavulanic acid (10 mg) was surrounded by ceftazidime (30 mg) and cefotaxime (5 mg) discs (Oxoid), with a distance of 25 to 30 mm from center to center. ESBL production was inferred by an extension of the margin of the inhibition zone of any disc towards the amoxicillin-clavulanic acid disc.

### 4.5. Identification of Carbapenemase-Coding and ESBL Genes

The DNA from the MDR isolates selected for PCR analysis was extracted using the Genomic DNA Purification Kit (Thermo Fisher Scientific, Waltham, MA, USA) as per the manufacturer’s recommendations. Using the appropriate primers created by Macrogen^®^ (Macrogen^®^, Madrid, Spain), PCR was used for screening the MDR isolates for the antimicrobial resistance genes *bla*_KPC_, *bla*_VIM_, *bla*_NDM_, *bla*_IMP_, *bla*_Oxa−48_, *bla*_CTX-M_*, bla*_SHV_, *bla*_TEM_, and *aac(6‘)-Ib* using the primers listed in Table S5. The following steps were used in the amplification reaction: initial 95 °C denaturation for 5 min; 30 cycles of denaturation at 95 °C for 40 s, annealing for 40 s, extension at 72 °C for 40 s; and a final extension step at 72 °C for 7 min. In order to electrophoretically separate the PCR products, a 1.5% agarose gel was stained with 3 µL of ethidium bromide. A 1000 bp DNA ladder (ThermoFisher Scientific, Waltham, MA, USA) was used to measure the size of DNA fragments, and agarose gel electrophoresis was used to analyze the amplified PCR data [8].

### 4.6. Phenotypic and Genotypic Analysis Using Heatmap Analysis:

The clonality of the MDR isolates was investigated based on the heatmap results. The Jaccard similarity measure was used for the hierarchical clustering of the isolates based on the phenotypic and genotypic data. This was computed using the online calculator (https://software.broadinstitute.org/morpheus/, accessed on 12 April 2023).

### 4.7. Statistical Analyses

Data analyses, including descriptive statistics, frequencies, and cross-tabulations, were carried out using IBM SPSS Statistics 20.0 software (SPSS, Inc., Chicago, IL, USA). Chi-Square and Fisher’s exact tests were used for categorical data analysis where appropriate. All tests of significance were two-tailed; a value of *p* < 0.05 was recognized as statistically significant.

## 5. Conclusions

This study updates the range of bacteria that cause FUO and emphasizes the high contribution of MDR and priority pathogens to this medical condition. The genetic basis of carbapenem resistance in MDR Gram-negative species was also explored. The findings of this study highlight the urgent need to effectively implement evidence-based antimicrobial stewardship and infection control programs. To help clinicians in tailoring existing empirical treatment guidelines for FUO complicated by resistant pathogens, we also provided a ranking of the tested antimicrobials with respect to their activity against Gram-positive and Gram-negative isolates recovered from patients suffering from this challenging medical condition.

## Data Availability

Data supporting the results is found in the main manuscript as well as in the Supplementary File.

## Supplementary Materials

The following supporting information can be downloaded at: https://www.mdpi.com/article/10.3390/antibiotics12081294/s1, Figure S1: Matrix showing the association between antimicrobial resistance phenotypes to the tested antimicrobial in *E. coli*; Figure S2: Matrix showing the association between antimicrobial resistance phenotypes to the tested antimicrobial agents in *K. pneumoniae*; Figure S3: Matrix showing the association between antimicrobial resistance phenotypes to the tested antimicrobial agents in *A. baumannii*; Figure S4: Matrix showing the association between antimicrobial resistance phenotypes to the tested antimicrobial agents in *P. aeruginosa;* Figure S5: Matrix showing the association between antimicrobial resistance phenotypes to the tested antimicrobial agents in *Proteus* sp.; Figure S6: Matrix showing the association between antimicrobial resistance phenotypes to the tested antimicrobial agents in *S. aureus*; Figure S7: Matrix showing the association between antimicrobial resistance phenotypes to the tested antimicrobial agents in *S. pneumoniae;* Figure S8: Matrix showing the association between antimicrobial resistance phenotypes to the tested antimicrobial agents in *Enterococci*; Table S1: The MICs of the tested antimicrobial agents, phenotypic and molecular analysis of Carbapenemase-encoding genes, ESBLs and *aac(6′)-Ib* of the tested isolates (*n* = 103); Table S2: *p*-values of the association between CR genes and the resistance to carbapenems in various species; Table S3: *p*-values of the association between ESBL genes and the resistance to β-lactams in various species; Table S4: *p*-values of the association between the *aac(6′)-Ib* gene and the resistance to aminoglycosides and fluoroquinolones in various species; Table S5: Primers used in this study, expected PCR product sizes, and annealing temperatures (Ta). References [76,77,78,79,80,81] are cited in the supplementary materials.
