# Supplementary material for: Phenotypic and Genotypic Analysis of Bacterial Pathogens Recovered from Patients Diagnosed with Fever of Unknown Origin in Egypt
Source: Antibiotics (Basel). 2023 Aug 7;12(8):1294. doi: 10.3390/antibiotics12081294 (PMC10451874; doi:10.3390/antibiotics12081294)
Supplement: Supplementary file 1 [file antibiotics-12-01294-s001.zip › antibiotics-2486688-supplementary.pdf]

Supplementary Material

	Cefaclor	Gentamicin10	Amikacin	Cotrimoxazole	Ciprofloxacin	Ofloxacin	Levofloxacin	Amoxacillin-Clavulanic acid	Ampicillin-sulbactam	Piperacillin tazobactam	Cefepime	Ceftriaxone	Cefotaxime	Ceftazidime	Cefoxitin	Meropenem	Imipenem	Doxycyclin	Colistin	Nitrofurantoin	Norfloxacin	Ampicillin
**Cefaclor**																						
**Gentamicin10**	0.073																					
**Amikacin**	0.503	0.003																				
**Cotrimoxazole**	0.186	0.45	0.561																			
**Ciprofloxacin**	0.002	0.023	0.222	0.006																		
**Ofloxacin**	0.001	0.032	0.249	0.004	0.001																	
**Levofloxacin**	0.002	0.148	0.054	0.009	0.001	0.001																
**Amoxacillin-Clavulanic acid**	0.007	0.195	0.46	0.001	0.034	0.026	0.045															
**Ampicillin-sulbactam**	0.003	0.041	0.561	0.571	0.001	0.001	0.009	0.245														
**Piperacillin tazobactam**	0.001	0.091	0.151	0.001	0.001	0.001	0.001	0.001	0.001													
**Cefepime**	0.001	0.01	0.155	0.186	0.012	0.009	0.002	0.007	0.065	0.001												
**Ceftriaxone**	0.001	0.155	0.114	0.011	0.001	0.001	0.001	0.018	0.045	0.001	0.001											
**Cefotaxime**	0.001	0.195	0.134	0.006	0.001	0.001	0.001	0.012	0.096	0.001	0.001	0.001										
**Ceftazidime**	0.001	0.029	0.098	0.019	0.002	0.001	0.001	0.005	0.068	0.001	0.001	0.001	0.001									
**Cefoxitin**	0.005	0.016	0.105	0.002	0.001	0.001	0.007	0.015	0.031	0.001	0.026	0.008	0.015	0.004								
**Meropenem**	0.032	0.005	0.001	0.035	0.001	0.001	0.001	0.024	0.141	0.001	0.032	0.018	0.024	0.013	0.001							
**Imipenem**	0.032	0.005	0.001	0.035	0.001	0.001	0.001	0.024	0.141	0.001	0.032	0.018	0.024	0.013	0.001	0.001						
**Doxycyclin**	0.142	0.525	0.596	0.01	0.131	0.089	0.084	0.005	0.387	0.177	0.047	0.148	0.089	0.225	0.199	0.237	0.237					
**Colistin**	0.358	0.555	0.786	0.493	0.686	0.702	0.67	0.62	0.507	0.637	0.358	0.402	0.38	0.424	0.732	0.358	0.358	0.732				
**Nitrofurantoin**	0.028	0.003	0.993	0.506	0.019	0.036	0.039	0.201	0.066	0.365	0.012	0.185	0.265	0.03	0.007	0.174	0.174	0.989	0.857			
**Norfloxacin**	0.001	0.001	0.651	0.297	0.001	0.001	0.001	0.062	0.014	0.001	0.001	0.01	0.025	0.001	0.001	0.006	0.006	0.088	0.705	0.001		
**Ampicillin**	NS	NS	NS	ND	ND	ND	ND	ND	ND	ND	ND	ND	ND	ND	ND	ND	ND	ND	ND	ND	ND	

Figure S1: Matrix showing the association between antimicrobial resistance phenotypes and the tested antimicrobial in *Escherichia coli*. Where appropriate, the *P*-values were calculated using the Chi-Square or Fisher's exact tests. Significant associations with *p*-values ≤ 0.05 are highlighted with a yellow color. ND, *p*-values could not be determined as all isolates showed the same phenotype (resistant or susceptible).

	Cefaclor	Gentamicin10	Amikacin	Cotrimoxazole	Ciprofloxacin	Ofloxacin	Levofloxacin	Amoxacillin-Clavulanic acid	Ampicillin-sulbactam	Piperacillin tazobactam	Cefepime	Ceftriaxone	Cefotaxime	Ceftazidime	Cefoxitin	Meropenem	Imipenem	Doxycyclin	Colistin	Nitrofurantoin	Norfloxacin	Ampicillin
**Cefaclor**																						
**Gentamicin10**	0.001																					
**Amikacin**	0.001	0.001																				
**Cotrimoxazole**	0.001	0.001	0.001																			
**Ciprofloxacin**	0.002	0.001	0.001	0.003																		
**Ofloxacin**	0.001	0.001	0.001	0.001	0.001																	
**Levofloxacin**	0.004	0.001	0.001	0.004	0.001	0.001																
**Amoxacillin-Clavulanic acid**	0.006	0.007	0.002	0.001	0.02	0.001	0.001															
**Ampicillin-sulbactam**	0.005	0.001	0.001	0.001	0.034	0.001	0.004	0.001														
**Piperacillin tazobactam**	0.002	0.001	0.001	0.001	0.001	0.001	0.001	0.001	0.006													
**Cefepime**	0.001	0.001	0.001	0.001	0.008	0.001	0.001	0.004	0.014	0.001												
**Ceftriaxone**	0.001	0.001	0.001	0.002	0.003	0.001	0.001	0.005	0.08	0.001	0.001											
**Cefotaxime**	0.001	0.014	0.001	0.01	0.159	0.001	0.004	0.005	0.001	0.001	0.001	0.001										
**Ceftazidime**	0.001	0.001	0.001	0.001	0.001	0.001	0.001	0.009	0.01	0.001	0.001	0.001	0.001									
**Cefoxitin**	0.008	0.001	0.001	0.004	0.015	0.001	0.001	0.001	0.012	0.001	0.002	0.017	0.004	0.003								
**Meropenem**	0.001	0.001	0.001	0.001	0.001	0.001	0.001	0.002	0.001	0.001	0.001	0.001	0.001	0.001	0.001							
**Imipenem**	0.001	0.001	0.001	0.001	0.001	0.001	0.001	0.001	0.001	0.001	0.001	0.001	0.001	0.001	0.001	0.001						
**Doxycyclin**	0.001	0.363	0.456	0.09	0.173	0.363	0.356	0.031	0.418	0.14	0.138	0.09	0.218	0.001	0.263	0.542	0.369					
**Colistin**	0.684	0.633	0.509	0.53	0.422	0.198	0.251	0.741	0.485	0.296	0.554	0.53	0.53	0.656	0.329	0.134	0.154	0.125				
**Nitrofurantoin**	0.001	0.013	0.009	0.01	0.142	0.084	0.759	0.868	0.108	0.076	0.001	0.008	0.001	0.034	0.276	0.001	0.003	0.036	0.504			
**Norfloxacin**	0.001	0.003	0.013	0.001	0.02	0.001	0.052	0.035	0.001	0.15	0.001	0.001	0.001	0.001	0.074	0.001	0.004	0.001	0.504	0.001		
**Ampicillin**	ND	ND	ND	ND	ND	ND	ND	ND	ND	ND	ND	ND	ND	ND	ND	ND	ND	ND	ND	ND	ND	

Figure S2: Matrix showing the association between antimicrobial resistance phenotypes and the tested antimicrobial in *Klebsiella pneumoniae*. Where appropriate, the *P*-values were calculated using the Chi-Square or Fisher's exact tests. Significant associations with *p*-values ≤ 0.05 are highlighted with a yellow color. ND, *p*-values could not be determined as all isolates showed the same phenotype (resistant or susceptible).

	Gentamicin10	Amikacin	Cotrimoxazole	Ciprofloxacin	Levofloxacin	Ampicillin-sulbactam	Piperacillin tazobactam	Cefepime	Ceftriaxone	Cefotaxime	Ceftazidime	Meropenem	Imipenem	Doxycycline	Colistin	Nitrofurantoin	Norfloxacin
**Gentamicin10**																	
**Amikacin**	0.001																
**Cotrimoxazole**	0.062	0.062															
**Ciprofloxacin**	0.031	0.031	0.046														
**Levofloxacin**	0.236	0.236	0.322	0.031													
**Ampicillin-sulbactam**	0.085	0.085	0.008	0.009	0.085												
**Piperacillin tazobactam**	0.004	0.004	0.008	0.009	0.085	0.027											
**Cefepime**	ND	ND	ND	ND	ND	ND	ND										
**Ceftriaxone**	0.192	0.192	0.231	0.077	0.192	0.115	0.115	ND									
**Cefotaxime**	ND	ND	ND	ND	ND	ND	ND	ND	ND								
**Ceftazidime**	0.085	0.085	0.008	0.009	0.085	0.001	0.027	ND	0.115	ND							
**Meropenem**	0.004	0.004	0.562	0.222	0.488	0.319	0.319	ND	0.115	ND	0.319						
**Imipenem**	0.014	0.014	0.676	0.711	0.4	0.592	0.408	ND	0.846	ND	0.592	0.052					
**Doxycycline**	0.03	0.03	0.404	0.28	0.578	0.14	0.14	ND	0.538	ND	0.14	0.14	0.359				
**Colistin**	ND	ND	ND	ND	ND	ND	ND	ND	ND	ND	ND	ND	ND	ND			
**Nitrofurantoin**	0.808	0.808	0.231	0.923	0.808	0.885	0.885	ND	0.962	ND	0.885	0.885	0.846	0.462	ND		
**Norfloxacin**	0.808	0.808	0.231	0.923	0.808	0.885	0.885	ND	0.962	ND	0.885	0.885	0.846	0.462	ND	0.038	

Figure S3: Matrix showing the association between antimicrobial resistance phenotypes and the tested antimicrobial in *Acinetobacter baumannii*. Where appropriate, the *P*-values were calculated using the Chi-Square or Fisher's exact tests. Significant associations with *p*-values ≤ 0.05 are highlighted with a yellow color. ND, *p*-values could not be determined as all isolates showed the same phenotype (resistant or susceptible).

	Gentamicin 10	Amikacin	Ciprofloxacin	Ofloxacin	Levofloxacin	Piperacillin tazobactam	Cefepime	Ceftazidime	Meropenem	Imipenem	Colistin
**Gentamicin 10**											
**Amikacin**	0.002										
**Ciprofloxacin**	0.051	0.015									
**Ofloxacin**	0.529	0.296	0.051								
**Levofloxacin**	0.051	0.015	0.001	0.051							
**Piperacillin tazobactam**	0.7	0.035	0.035	0.7	0.035						
**Cefepime**	0.385	0.5	0.5	0.385	0.5	0.33					
**Ceftazidime**	0.175	0.035	0.035	0.594	0.035	0.21	0.67				
**Meropenem**	0.7	0.035	0.035	0.07	0.035	0.001	0.33	0.21			
**Imipenem**	0.028	0.133	0.133	0.238	0.133	0.005	0.231	0.545	0.005		
**Colistin**	0.571	0.5	0.5	0.429	0.5	0.714	0.786	0.286	0.714	0.357	

Figure S4: Matrix showing the association between antimicrobial resistance phenotypes and the tested antimicrobial in *Pseudomonas aeruginosa*. Where appropriate, the *P*-values were calculated using the Chi-Square or Fisher's exact tests. Significant associations with *p*-values ≤ 0.05 are highlighted with a yellow color. ND, *p*-values could not be determined as all isolates showed the same phenotype (resistant or susceptible).

	Cefaclor	Gentamicin 10	Amikacin	Cotrimoxazole	Ciprofloxacin	Ofloxacin	Levofloxacin	Amoxacillin-Clavulanic acid	Ampicillin-sulbactam	Piperacillin tazobactam	Cefepime	Ceftriaxone	Cefotaxime	Ceftazidime	Cefoxitin	Meropenem	Imipenem	Doxycycline
**Cefaclor**																		
**Gentamicin 10**	0.667																	
**Amikacin**	ND	ND																
**Cotrimoxazole**	0.667	0.333	ND															
**Ciprofloxacin**	ND	ND	ND	ND														
**Ofloxacin**	0.667	0.667	ND	0.667	ND													
**Levofloxacin**	0.667	0.667	ND	0.667	ND	0.333												
**xacillin-Clavulanic acid**	0.333	0.667	ND	0.667	ND	0.667	0.667											
**Ampicillin-sulbactam**	0.333	0.667	ND	0.667	ND	0.667	0.667	0.333										
**Piperacillin tazobactam**	0.667	0.667	ND	0.667	ND	0.333	0.333	0.667	0.667									
**Cefepime**	ND	ND	ND	ND	ND	ND	ND	ND	ND	ND								
**Ceftriaxone**	ND	ND	ND	ND	ND	ND	ND	ND	ND	ND	ND							
**Cefotaxime**	ND	ND	ND	ND	ND	ND	ND	ND	ND	ND	ND	ND						
**Ceftazidime**	0.667	0.667	ND	0.667	ND	0.333	0.333	0.667	0.667	0.333	ND	ND	ND					
**Cefoxitin**	0.667	0.333	ND	0.333	ND	0.667	0.667	0.667	0.667	0.667	ND	ND	ND	0.667				
**Meropenem**	ND	ND	ND	ND	ND	ND	ND	ND	ND	ND	ND	ND	ND	ND	ND			
**Imipenem**	ND	ND	ND	ND	ND	ND	ND	ND	ND	ND	ND	ND	ND	ND	ND	ND		
**Doxycycline**	ND	ND	ND	ND	ND	ND	ND	ND	ND	ND	ND	ND	ND	ND	ND	ND	ND	

Figure S5: Matrix showing the association between antimicrobial resistance phenotypes and the tested antimicrobial in *Proteus* sp. Where appropriate, the *P*-values were calculated using the Chi-Square or Fisher's exact tests. Significant associations with *p*-values ≤ 0.05 are highlighted with a yellow color. ND, *p*-values could not be determined as all isolates showed the same phenotype (resistant or susceptible).

	Penicillin	Cefoxitin	Trimethoprim/Sulfamethoxazole	Clindamycin	Erythromycin	Azithromycin	Vancomycin	Doxycycline	Rifampicin	Linezolid	Ciprofloxacin	Ofloxacin	Levofloxacin	Gentamicin 10
**Penicillin**														
**Cefoxitin**	0.111													
**Trimethoprim/Sulfamethoxazole**	0.913	0.652												
**Clindamycin**	0.676	0.154	0.826											
**Erythromycin**	0.739	0.611	0.478	0.037										
**Azithromycin**	0.692	0.51	0.435	0.024	0.001									
**Vancomycin**	ND	ND	ND	ND	ND	ND								
**Doxycycline**	0.676	0.154	0.826	0.562	0.329	0.404	ND							
**Rifampicin**	0.913	0.652	0.957	0.174	0.478	0.435	ND	0.174						
**Linezolid**	ND	ND	ND	ND	ND	ND	ND	ND	ND					
**Ciprofloxacin**	0.308	0.195	0.435	0.596	0.407	0.552	ND	0.596	0.435	ND				
**Ofloxacin**	0.36	0.069	0.391	0.517	0.567	0.637	ND	0.517	0.391	ND	0.001			
**Levofloxacin**	0.415	0.013	0.348	0.435	0.611	0.49	ND	0.435	0.348	ND	0.003	0.001		
**Gentamicin 10**	0.415	0.013	0.348	0.435	0.278	0.184	ND	0.435	0.348	ND	0.184	0.367	0.253	

Figure S6: Matrix showing the association between antimicrobial resistance phenotypes and the tested antimicrobial in *Staphylococcus aureus*. Where appropriate, the *P*-values were calculated using the Chi-Square or Fisher's exact tests. Significant associations with *p*-values ≤ 0.05 are highlighted with a yellow color. ND, *p*-values could not be determined as all isolates showed the same phenotype (resistant or susceptible).

	Cefoxitin	Trimethoprim/Sulfamethoxazole	Clindamycin	Erythromycin	Azithromycin	Vancomycin	Doxycycline	Rifampicin	Linezolid	Ciprofloxacin	Ofloxacin	Levofloxacin	Chloamphenicol	Imipenem	Cefotaxime
Cefoxitin															
Trimethoprim/Sulfamethoxazole	0.714														
Clindamycin	0.429	0.286													
Erythromycin	0.571	0.714	0.371												
Azithromycin	0.571	0.714	0.114	0.114											
Vancomycin	ND	ND	ND	ND	ND										
Doxycycline	0.429	0.714	0.629	0.114	0.371	ND									
Rifampicin	0.857	0.714	0.571	0.571	0.429	ND	0.571								
Linezolid	ND	ND	ND	ND	ND	ND	ND	ND							
Ciprofloxacin	0.286	0.524	0.714	0.714	0.286	ND	0.714	0.714	ND						
Ofloxacin	ND	ND	ND	ND	ND	ND	ND	ND	ND	ND					
Levofloxacin	ND	ND	ND	ND	ND	ND	ND	ND	ND	ND	ND				
Chloamphenicol	ND	ND	ND	ND	ND	ND	ND	ND	ND	ND	ND	ND			
Imipenem	ND	ND	ND	ND	ND	ND	ND	ND	ND	ND	ND	ND	ND		
Cefotaxime	ND	ND	ND	ND	ND	ND	ND	ND	ND	ND	ND	ND	ND	ND	

Figure S7: Matrix showing the association between antimicrobial resistance phenotypes and the tested antimicrobial in *Streptococcus pneumoniae*. Where appropriate, the *P*-values were calculated using the Chi-Square or Fisher's exact tests. Significant associations with *p*-values ≤ 0.05 are highlighted with a yellow color. ND, *p*-values could not be determined as all isolates showed the same phenotype (resistant or susceptible).

	Penicillin	Erythromycin	Azithromycin	Vancomycin	Doxycycline	Rifampicin	Linezolid	Ciprofloxacin	Ofloxacin	Levofloxacin	Gentamicin 120	Chloamphenicol	Ampicillin
**Penicillin**													
**Erythromycin**	0.116												
**Azithromycin**	0.116	0.001											
**Vancomycin**	ND	ND	ND										
**Doxycycline**	0.286	0.45	0.45	ND									
**Rifampicin**	0.613	0.55	0.55	ND	0.418								
**Linezolid**	ND	ND	ND	ND	ND	ND							
**Ciprofloxacin**	0.618	0.307	0.307	ND	0.328	0.672	ND						
**Ofloxacin**	0.525	0.564	0.564	ND	0.541	0.459	ND	0.016					
**Levofloxacin**	0.475	0.564	0.564	ND	0.186	0.459	ND	0.016	0.002				
**Gentamicin 120**	0.387	0.223	0.223	ND	0.157	0.418	ND	0.265	0.015	0.015			
**Chloamphenicol**	0.496	0.649	0.649	ND	0.163	0.57	ND	0.396	0.038	0.038	0.011		
**Ampicillin**	0.022	0.601	0.601	ND	0.284	0.052	ND	0.468	0.699	0.313	0.144	0.231	

Figure S8: Matrix showing the association between antimicrobial resistance phenotypes and the tested antimicrobial in *Enterococci*. Where appropriate, the *P*-values were calculated using the Chi-Square or Fisher's exact tests. Significant associations with *p*-values ≤ 0.05 are highlighted with a yellow color. ND, *p*-values could not be determined as all isolates showed the same phenotype (resistant or susceptible).

Table S1: The MICs of the tested antimicrobial agents, phenotypic and molecular analysis of Carbapenemase-encoding genes, ESBLs, and *aac(6′)Ib* of the tested isolates (n = 103):

Bacterial species	Resistance genes			No. of isolates	MIC range			
	ESBLs	CPases	*aac(6′)-Ib*		IMP	CTX	FEP	CIP
E. coli	$bla_{\text{CTX-M}}$, bla_{TEM}	$bla_{\text{OXA-48}}$	+	15	16–265	24–512	24–512	32–256
	$bla_{\text{CTX-M}}$, bla_{TEM}, bla_{SHV}	$bla_{\text{OXA-48}}$	+	7	16–128	32–512	256	32–128
	$bla_{\text{CTX-M}}$, bla_{TEM}	____	+	2	ND	32	256	32
	$bla_{\text{CTX-M}}$, bla_{TEM}, bla_{SHV}	bla_{IMP}	+	1	ND	32	32	32
	$bla_{\text{CTX-M}}$, bla_{TEM}	bla_{VIM}	+	1	ND	512	256	128
K. pneumoniae	$bla_{\text{CTX-M}}$, bla_{TEM}, bla_{SHV}	$bla_{\text{OXA-48}}$	-	3	32	32–512	48–256	32–128
	$bla_{\text{CTX-M}}$, bla_{TEM}, bla_{SHV}	$bla_{\text{OXA-48}}$	+	24	12–255	32–512	48–256	32–127
	$bla_{\text{CTX-M}}$, bla_{TEM}	$bla_{\text{OXA-48}}$	-	5	32–64	512	64–256	128
	$bla_{\text{CTX-M}}$, bla_{TEM}	$bla_{\text{OXA-48}}$	+	5	64–128	24–512	32–256	32–256
	$bla_{\text{CTX-M}}$, bla_{TEM}	$bla_{\text{OXA-48}}$, bla_{VIM}, bla_{IMP}	-	1	ND	32	256	6
	$bla_{\text{CTX-M}}$, bla_{TEM}, bla_{SHV}	$bla_{\text{OXA-48}}$, bla_{IMP}	+	6	64–256	256–512	128–256	64–128
	$bla_{\text{CTX-M}}$, bla_{TEM}, bla_{SHV}	$bla_{\text{OXA-48}}$, bla_{IMP}	-	1	16	24	256	32
	$bla_{\text{CTX-M}}$, bla_{TEM}	$bla_{\text{OXA-48}}$, bla_{IMP}	+	2	32	32–512	64–256	8_16
	$bla_{\text{CTX-M}}$, bla_{TEM}	___	+	1	ND	512	128	256
	$bla_{\text{CTX-M}}$, bla_{TEM}, bla_{SHV}	___	-	2	ND	32	256	32
	$bla_{\text{CTX-M}}$, bla_{TEM}, bla_{SHV}	$bla_{\text{OXA-48}}$, bla_{VIM}, bla_{IMP}	+	1	ND	512	256	128
	$bla_{\text{CTX-M}}$, bla_{TEM}, bla_{SHV}	____	+	1	64	512	128	128
	$bla_{\text{CTX-M}}$, bla_{TEM}, bla_{SHV}	$bla_{\text{OXA-48}}$	+	1	ND	256	64	64
A. baumannii	$bla_{\text{CTX-M}}$, bla_{TEM}, bla_{SHV}	$bla_{\text{OXA-48}}$	+	2	32–128	32–512	256	32–128
	$bla_{\text{CTX-M}}$, bla_{TEM}	___	+	3	16–32	512	256–512	8–128
	$bla_{\text{CTX-M}}$, bla_{TEM}	$bla_{\text{OXA-48}}$	+	2	32–256	512	256	64–512
	bla_{TEM}	$bla_{\text{OXA-48}}$	+	3	32–128	512	256	32–128
	$bla_{\text{CTX-M}}$, bla_{TEM}	bla_{VIM}	-	1	ND	32	64	24

	*bla*CTX-M, *bla*TEM	*bla*OXA-48, *bla*VIM,	+	1	32	32	256	32
	*bla*TEM	*bla*OXA-48	-	1	32	32	256	32
	*bla*CTX-M, *bla*TEM, *bla*SHV	*bla*VIM	+	1	32	32	256	32
	*bla*CTX-M, *bla*TEM	*bla*OXA-48	-	1	256	512	512	32
	*bla*CTX-M	*bla*OXA-48	+	1	256	512	256	64
	*bla*CTX-M, *bla*TEM, *bla*SHV	*bla*OXA-48, *bla*NDM	+	1	32	32	256	32
	*bla*CTX-M	*bla*OXA-48	+	1	256	512	256	64
	*bla*TEM	*bla*OXA-48, *bla*VIM, *bla*KPC	+	1	256	512	256	64
	*bla*TEM	*bla*OXA-48, *bla*VIM	+	1	32	32	256	32
P. aeruginosa	*bla*CTX-M, *bla*TEM	——	+	2	128–256	512	512	64–128
	*bla*CTX-M, *bla*TEM, *bla*SHV	*bla*VIM	+	1	512	512	512	256
	*bla*CTX-M, *bla*TEM	*bla*OXA-48	+	1	16	512	512	8

Table S2. P-values of the association between CR genes and the resistance to carbapenems in various species

Bacterial Species	*Genes*	Meropenem	Imipenem
	*bla*KPC	ND	ND
	*bla*VIM	1	1
E. coli	*bla*NDM	ND	ND
	*bla*OXA-48	0.614	0.136
	*bla*IMP	1	1
	*bla*KPC	ND	ND
	*bla*VIM	0.026	0.011
K. pneumoniae	*bla*NDM	ND	ND
	*bla*OXA-48	0.071	0.308
	*bla*IMP	1	0.592
	*bla*KPC	ND	1
	*bla*VIM	ND	0.25
A. baumannii	*bla*NDM	ND	1
	*bla*OXA-48	ND	0.3
	*bla*IMP	ND	ND
	*bla*KPC	ND	ND
	*bla*VIM	ND	ND
P. aeruginosa	*bla*NDM	ND	ND
	*bla*OXA-48	ND	ND
	*bla*IMP	ND	ND

P-values were calculated by Chi-Square or Fisher's exact tests where appropriate. Significant associations with *p*-values ≤ 0.05 are highlighted with a yellow color. ND, *P*-values could not be calculated as all isolates showed the same resistance phenotype (resistant or sensitive) or all isolates had the same genotype (the gene was present or absent in all).

Table S3. P-values of the association between ESBL genes and the resistance to β-lactams in various species

Bacterial Species	*Gene*	CEC	AMP	FOX	AMC	SAM	TPZ	FEP	CRO	CTX	CAZ
E. coli	***bla***$_{\text{SHV}}$	**ND**	**1**	**1**	**1**	**0.529**	**0.628**	**ND**	**ND**	**ND**	**ND**
	bla$_{\text{CTX-M}}$	**ND**	**ND**	**ND**	**ND**	**ND**	**ND**	**ND**	**ND**	**ND**	**ND**
	bla$_{\text{TEM}}$	**ND**	**ND**	**ND**	**ND**	**ND**	**ND**	**ND**	**ND**	**ND**	**ND**
K. pneumoniae	***bla***$_{\text{SHV}}$	**ND**	**ND**	**0.567**	**ND**	**0.145**	**1**	**ND**	**ND**	**ND**	**ND**
	bla$_{\text{CTX-M}}$	**ND**	**ND**	**ND**	**ND**	**ND**	**ND**	**ND**	**ND**	**ND**	**ND**
	bla$_{\text{TEM}}$	**ND**	**ND**	**ND**	**ND**	**ND**	**ND**	**ND**	**ND**	**ND**	**ND**
A. baumannii	***bla***$_{\text{SHV}}$					**ND**	**1**	**ND**	**ND**	**ND**	**ND**
	bla$_{\text{CTX-M}}$					**ND**	**1**	**ND**	**ND**	**ND**	**ND**
	bla$_{\text{TEM}}$					**ND**	**ND**	**ND**	**ND**	**ND**	**ND**
P. aeruginosa	***bla***$_{\text{SHV}}$						**ND**	**ND**			**ND**
	bla$_{\text{CTX-M}}$						**ND**	**ND**			**ND**
	bla$_{\text{TEM}}$						**ND**	**ND**			**ND**

P-values were calculated by Chi-Square or Fisher's exact tests where appropriate. Black cells correspond to untested antimicrobial agents. ND, *P*-values could not be calculated as all isolates showed the same resistance phenotype (resistant or sensitive) or all isolates had the same genotype (the gene was present or absent in all). AMP, ampicillin; AMC, amoxacillin/clavulanic acid; CAZ, ceftazidime; CEC, cefaclor; CRO, ceftriaxone; CTX, cefotaxime; FEP, Cefepime; FOX, cefoxitin; SAM, ampicillin/sulbactam; TPZ, piperacillin/tazobactam.

Table S4. P-values of the association between the *aac(6')-Ib* gene and the resistance to aminoglycosides and fluoroquinolones in various species

Bacterial species	Gentamicin 10	Amikacin	Ciprofloxacin	Ofloxacin	Levofloxacin
E. coli	ND	ND	ND	ND	ND
K. pneumoniae	0.677	0.244	1	0.07	0.07
A. baumannii	0.046	0.15	ND		0.404
P. aeruginosa	ND	ND	ND	ND	ND

P-values were calculated by Chi-Square or Fisher's exact tests where appropriate. Black cells correspond to untested antimicrobial agents, while significant associations with *p*-values ≤ 0.05 are highlighted with a yellow color. ND, *P*-values could not be calculated as all isolates showed the same resistance phenotype (resistant or sensitive) or all isolates had the same genotype (the gene was present or absent in all).

Table S5: Primers used in this study, expected PCR product sizes, and annealing temperatures (Ta)

PCR reaction	Gene	Primer	Primer sequence (5′ → 3′)	Expected PCR product size (bp)	T_a (°C)	References
Multiplex	*bla*$_{KPC}$	P_f	TGTCACTGTATCGCCGTC	1011	50	[1]
		P_r	CTCAGTGCTCTACAGAAAACC			
	bla$_{NDM}$	P_f	GGTTTGGCGATCTGGTTTTC	621		[2]
		P_r	CGGAATGGCTCATCACGAT			
Multiplex	*bla*$_{VIM}$	P_f	TCTACATGACCGCGTCTGTC	748	50	[3]
		P_r	TGTGCTTTGACAACGTTCGC			
	bla$_{OXA-48}$	P_f	GCGTGGTTAAGGATGAACAC	438		[1]
		P_r	CATCAAGTTCAACCCAACCG			
Monoplex	*bla*$_{IMP}$	P_f	CTACCGCAGCAGAGTCTTTG	587	50	[4]
		P_r	AACCAGTTTTGCCTTACCAT			
Multiplex	*aac(6′)-Ib*	P_f	TTGCGATGCTCTATGAGTGG	358	49	[5]
		P_r	CGTTTGGATCTTGGTGACCT			
	bla$_{SHV}$	P_f	GGTTATGCGTTATATTCGCC	867		[6]
		P_r	TTAGCGTTGCCAGTGCTC			
Multiplex	*bla*$_{CTX-M}$	P_f	CGCTTTGCGATGTGCAG	550	51	[7]
		P_r	ACCGCGATATCGTTGGT			
	bla$_{TEM}$	P_f	ATGAGTATTCAACATTTCCG	867		[6]
		P_r	CTGACAGTTACCAATGCTTA			

Abbreviations: P_f, forward primer; P_r, reverse primer; T_a, annealing temperature.
